# Epidemiology Characteristics, Methodological Assessment and Reporting of Statistical Analysis of Network Meta-Analyses in the Field of Cancer

**DOI:** 10.1038/srep37208

**Published:** 2016-11-16

**Authors:** Long Ge, Jin-hui Tian, Xiu-xia Li, Fujian Song, Lun Li, Jun Zhang, Ge Li, Gai-qin Pei, Xia Qiu, Ke-hu Yang

**Affiliations:** 1 The First Clinical Medical College of Lanzhou University, Lanzhou 730000, China; 2 Evidence-based Medicine Center of Lanzhou University, Lanzhou 730000, China; 3 Key Laboratory of Evidence-based Medicine and Knowledge Translation of Gansu Province, Lanzhou 730000, China; 4 Norwich Medical School, Faculty of Medicine and Health Science, University of East Anglia, Norwich, Norfolk, UK; 5 Department of Breast-Thyroid Surgery, The Second Xiangya Hospital of Central South University, Changsha 410000, China; 6 School of Basic Medical Sciences, Gansu University of Traditional Chinese Medicine, Lanzhou 730000, China; 7 School of Chinese Medicine, Tianjin University of Traditional Chinese Medicine, Tianjin 300193, China; 8 The Second Clinical Medical College of Lanzhou University, Lanzhou 730000, China

## Abstract

Because of the methodological complexity of network meta-analyses (NMAs), NMAs may be more vulnerable to methodological risks than conventional pair-wise meta-analysis. Our study aims to investigate epidemiology characteristics, conduction of literature search, methodological quality and reporting of statistical analysis process in the field of cancer based on PRISMA extension statement and modified AMSTAR checklist. We identified and included 102 NMAs in the field of cancer. 61 NMAs were conducted using a Bayesian framework. Of them, more than half of NMAs did not report assessment of convergence (60.66%). Inconsistency was assessed in 27.87% of NMAs. Assessment of heterogeneity in traditional meta-analyses was more common (42.62%) than in NMAs (6.56%). Most of NMAs did not report assessment of similarity (86.89%) and did not used GRADE tool to assess quality of evidence (95.08%). 43 NMAs were adjusted indirect comparisons, the methods used were described in 53.49% NMAs. Only 4.65% NMAs described the details of handling of multi group trials and 6.98% described the methods of similarity assessment. The median total AMSTAR-score was 8.00 (IQR: 6.00–8.25). Methodological quality and reporting of statistical analysis did not substantially differ by selected general characteristics. Overall, the quality of NMAs in the field of cancer was generally acceptable.

Cancer is a leading cause of death worldwide and the total number of global cancer deaths is projected to increase by 45% from 7.9 million in 2007 to 11.5 million in 2030^1^. For patients suffering from cancer, healthcare interventions aim to cure or considerably prolong the life of patients and to ensure the best possible quality of life for cancer survivors^1^. Treatment decisions should be based on evidence of the existing most effective treatment given available resources. High quality systematic reviews/meta-analyses of randomized controlled trials (RCTs) can provide the most valid evidence^2^. However, conventional meta-analysis becomes inadequate when there are no head-to-head trials comparing alternative interventions, or when more than two interventions need to be compared simultaneously^3^. For example, although there are trials directly comparing each of the newer antineoplastic agents with the current standard treatment (or placebo) for patients with neoplasm, there are no trials that directly compared different newer antineoplastic agents. Another example is a lack of direct comparison of 19 different chemotherapy regimens that are currently available for the treatment of advanced pancreatic cancer^4^.

Network meta-analyses (NMAs), as a generalization of pairwise meta-analysis, is becoming increasingly popular^5^,^6^,^7^,^8^. In the absence of or insufficient head-to-head comparisons of competing interventions of interest, NMAs using indirect treatment comparison analyses can provide useful evidence to inform health-care decision making. Even when evidence from direct comparisons are available, combining them with indirect estimates in a mixed treatment comparison may yield more refined estimates^8^,^9^. Formally, NMAs can be defined as a statistical combination of all available evidence for an outcome from several studies across multiple treatment to generate estimates of pairwise comparisons of each intervention to every other intervention within a network^10^. It has been considered that NMAs would be the next generation evidence synthesis toolkit which, when properly applied, could serve decision-making better than the conventional pair-wise meta-analysis^11^. However, NMAs are subject to similar methodological risks as standard pairwise systematic reviews. Because of its methodological complexity, it is probable that NMAs may be more vulnerable to such risks^12^. Therefore, it is important to assess the quality of published NMAs before their results are implemented into clinical or public health practice.

Previous studies have examined methodological problems in published indirect comparisons and NMAs, especially regarding reporting quality of statistical analysis^12^,^13^,^14^,^15^. It was concluded that the key methodological components of the NMAs process were often inadequately reported in published NMAs^12^. Currently, there are 30 tools available to assess the methodological quality of systematic reviews or meta-analyses^16^. To the best of our knowledge, no standard tool has been developed currently to assess the methodological quality of NMAs. AMSTAR (a measurement tool to assess the methodological quality of systematic reviews) tool is probably the most commonly used quality assessment tool for systematic reviews, which has been proven with good reliability, validity, and responsibility^17^,^18^,^19^.

The objective of this study is to conduct a methodological review of published NMAs in the field of cancer, summarise their characteristics, methodological quality, and reporting of key statistical analysis process. We also aim to compare the methodological quality and reporting of statistical analysis by selected general characteristics.

## Results

### Search results

**Figure 1:**
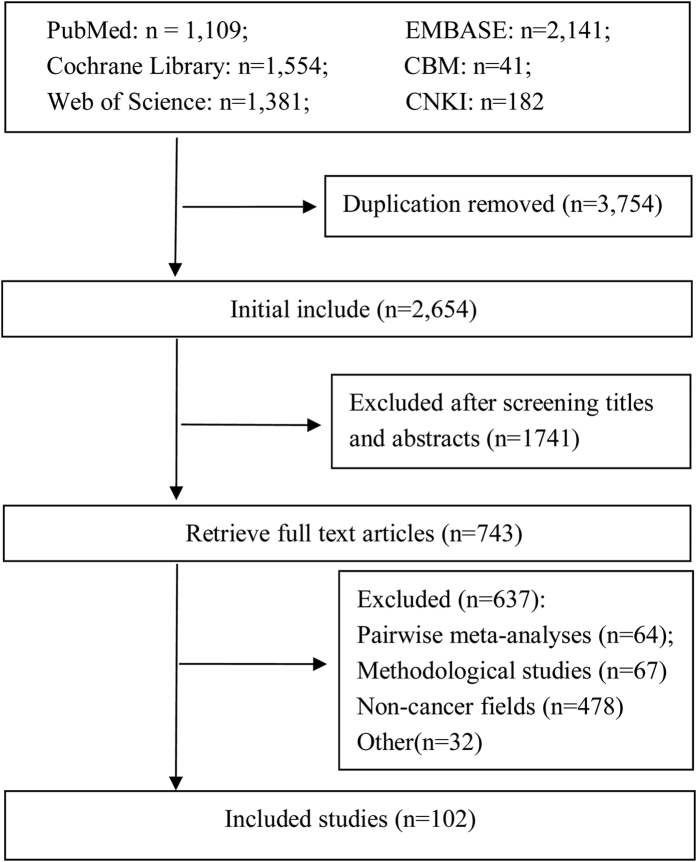
The details of literature selection.

Initial literature search retrieved 6,408 citations. Of them, 3,754 citations were duplicates, so 2,654 citations were sent for further screening. Based on titles and abstracts, 1,741 citations were excluded. Then 637 articles were excluded based on reading full-texts, for reasons including: traditional pair-wise meta analysis (n = 64), methodological studies (n = 67), NMAs not related to cancer (n = 478), abstracts/letters/editorials/correspondences (n = 26), cost-effectiveness reviews (n = 6). Finally, 102 NMAs in the field of cancer were included (Fig. 1), including 92 published in English and 10 in Chinese. A list of included NMAs could be found in Appendix 1.

### General characteristics of included NMAs

**Figure 2:**
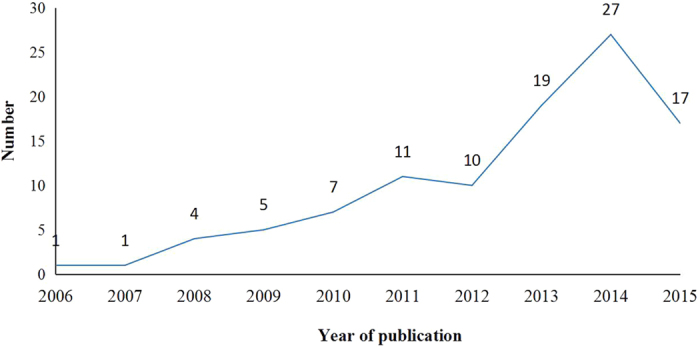
The trend of year of publications.

**Figure 3:**
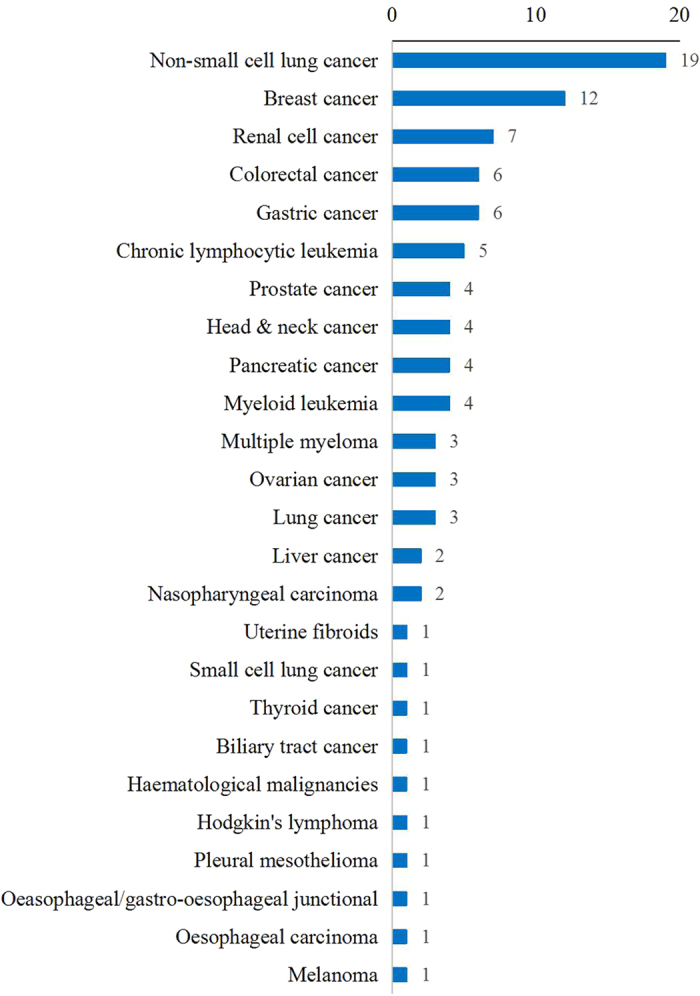
Categories of disease of included NMAs.

**Figure 4:**
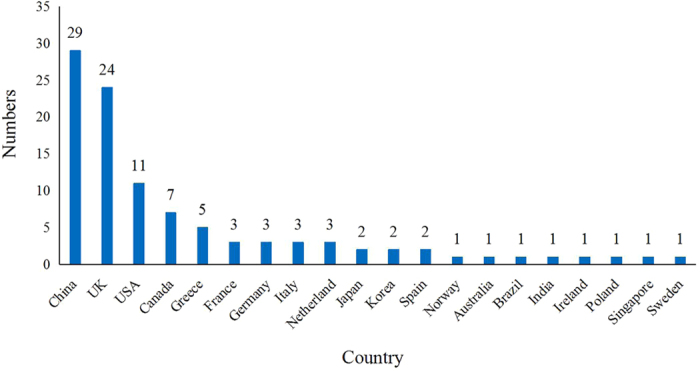
Countries of included NMAs.

The first NMA in the field of cancer was published in 2006^20^. The number of published NMAs increased slowly until 2010, and then increased quickly. 43.14% (44/102) of the included NMAs were published since 2014 (Fig. 2). 98 NMAs involved 24 kinds of cancer, although 4 NMAs did not specify types of cancer. Non-small cell lung cancer (19/102, 18.63%) and breast cancer (12/102, 11.76%) were the most or secondly common type of cancer studies in the included NMAs (Fig. 3). NMAs were often performed by researchers based in China (29/102, 28.43%), UK (24/102, 23.53%), and USA (11/102, 10.78%) (Fig. 4). 99 NMAs were published in 60 different journals and 3 NMAs were doctorate dissertations. 85.30% (87/102) of NMAs were indexed by Science Citation Index (SCI) and 31.37% (32/102) were published in journals with high impact factors. According to 56 NMAs (54.90%) that reported dates of manuscript reception and acceptation, the median publishing period was 101 days (IQR: 47–187 days). According to 60 NMAs (58.82%) with information on funding source, 46 NMAs (45.10%) received funding support. The median number of interventions assessed per NMA was five (IQR: 3–9). The median number of trials included per NMAs was 12 (IQR: 7–23), and the median number of patients included in NMAs was 3,605 (IQR: 1,950–7,564). The main characteristics of the included NMAs were shown in Table 1. A more detailed characteristics and reporting of statistical analysis process could be found in Appendix 2.

### Reporting of literature search

Thirteen NMAs did not report any information on literature search, whereas one NMAs was conducted based on previous meta-analyses without additional searching. 98.90% (88/89) NMAs searched only English databases. The median number of Chinese databases searched was 5 (IQR: 3–6), and it was 3 (IQR: 3–4) for English databases. 22.50% (20/89) NMAs reported the search strategy, and the median number of search strategies reported was 2 (IQR: 1–3). 27.00% (24/89) NMAs searched previous published meta-analyses as a supplemental literature search. Other supplemental literature search methods included reference list checking, clinical trial registration platform, conference abstracts or web sites, and google engine (Table 2). PubMed/MEDLINE was the most common single database searched, and it was often combined with a search of Cochrane Library. The details of databases searched were showed in Table 3.

### Reporting of statistical analysis processes

Sixty-one (59.80%) NMAs were conducted using a Bayesian framework (2 reviews are adjusted indirect comparisons). 43 reviews were adjusted indirect comparisons (2 adjusted indirect comparisons use Bayesian framework).

For NMAs using a Bayesian framework, more than half also included traditional meta-analyses (42/61, 68.85%). The majority of NMAs reported summary measures (57/61, 93.44%). 75.41% (46/61) NMAs reported the model used. Of the 24 (24/61, 39.34%) NMAs that tested model fit, the most common method was the use of deviance information criterion (15/24, 62.50%). The majority of NMAs (40/61, 65.57%) did not make their code available to journal readers. 16 (26.23%) NMAs provided the model source cited, however, it was unclear for the details of model used. 91.80% (56/61) did not report whether there was an adjustment for multiple arms. Half of NMAs (31/61, 50.82%) specified the prior distributions used, and the most common prior used was non-informative prior (18/31, 58.06%). Only 4 (6.56%) NMAs performed pre-specified sensitivity analyses. More than half of NMAs did not report assessment of convergence (37/61, 60.66%) and sensitivity analyses performed (37/61, 60.66%). Inconsistency was assessed in 27.87% of NMAs. Assessment of heterogeneity in traditional meta-analyses was more common (26/61, 42.62%) than in NMAs (4/61, 6.56%). Most of the included NMAs did not report assessment of similarity (53/61, 86.89%), publication or reporting bias (60/61, 98.36%), subgroup analyses or meta-regression performed (49/61, 80.33%), and whether GRADE tool was used to assess quality of evidence (58/61, 95.08%). NMAs published in journals with higher impact factors more often provided model code (57.69% versus 23.08%, p = 0.012) and assessed the heterogeneity of NMAs (0% versus 15.38%, p = 0.039). Based on the median division of the number of included NMAs, we chose December 31st 2013 as cut-off point. NMAs published prior to December 31st 2013 more often reported models used (89.66% versus 62.50%, p = 0.015), model code used (48.28% versus 21.88%, p = 0.032), and assessment of heterogeneity of NMAs (13.79% versus 0%, p = 0.031). Other results did not differ by journal impact factor or year of publication. The more details of statistical reporting in Bayesian NMAs was showed in Table 4.

For adjusted indirect comparisons, the majority of NMAs (42/43, 97.67%) also conducted traditional meta-analyses and 53.49% (23/43) adjusted indirect comparisons were performed using methods described by Bucher^21^. 58.14% (25/43) assessed the heterogeneity of direct comparisons, but none of NMAs assessed the heterogeneity of indirect comparisons. Only two (4.65%) NMAs described the details of handling of multi group trials and three (6.98%) described the methods of similarity assessment. Most of NMAs did not report whether sensitivity analyses were performed (38/43, 88.37%) and whether subgroup analyses or meta-regression were performed (34/43, 79.07%). These results did not differ by journal quality or year of publication. The details of statistical reporting for adjusted indirect comparisons was showed in Table 5.

### Methodological quality assessment

**Figure 5:**
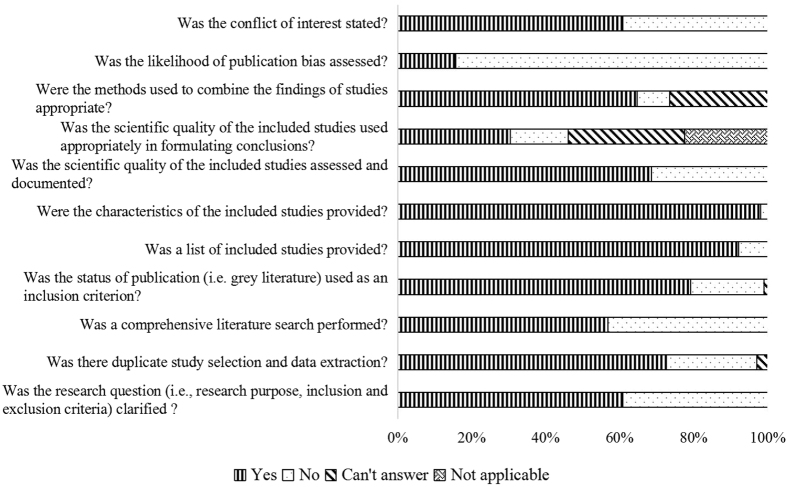
The results of methodological quality assessment.

The results of methodological quality assessment based on modified AMSTAR checklist were presented in Fig. 5. The median total score was 8.00 (IQR: 6.00–8.25). Approximately half of the included NMAs did not perform a comprehensive literature search (Item 3, 42.31%). More than half of NMAs (69.61%) did not consider the scientific quality of the included studies in formulating conclusions, and 84.31% NMAs did not assess the likelihood of publication bias.

Table 6 presented the results of stratified analyses of methodological quality assessment. NMAs published in journals with higher impact factors more often performed a comprehensive literature search (78.13% versus 45.45%, p = 0.002), reported appropriate methods used to combine the findings of studies (81.25% versus 58.18%, p = 0.019), and assessed the likelihood of publication bias (25.00% versus 5.45%, p = 0.017). NMAs published after December 31st 2013 more often assessed the scientific quality of the included studies (86.36% versus 55.17%, p = 0.001) and considered the scientific quality in formulating conclusions (43.18% versus 20.69%, p = 0.015). Most of these items did not differ between funding support and non-funding support. NMAs published in China more often reported two independent reviewers for study selection and data extraction (89.66% versus 65.75%, p = 0.015), assessed the scientific quality of the included studies (86.21% versus 61.64%, p = 0.016) and considered the scientific quality in formulating conclusions (68.97% versus 15.07%, p = 0.000). Moreover, Bayesian NMAs more often reported two independent reviewers for study selection and data extraction (81.97% versus 60.47%, p = 0.015), performed a comprehensive literature search (70.49% versus 39.53%, p = 0.002), considered the status of publication (i.e. grey literature) used as an inclusion criterion (86.89% versus 67.44%, p = 0.017), and assessed the scientific quality of the included studies (78.69% versus 55.81%, p = 0.013).

Table 7 presented the association of total AMSTAR-score and selected general characteristics. Although the AMSTAR-score of NMAs published in China was higher than NMAs published in others (p = 0.023), there were no significant differences between AMSTAR-score and different countries (p = 0.465). The differences were not significant between AMSTAR-score and other selected general characteristics.

## Discussion

We identified 102 NMAs involving 24 kinds of cancer. Methodological quality and statistical reporting were assessed based on PRISMA extension statement and modified AMSTAR checklist. In addition, we also assessed the conduct of literature search in the included NMAs. Some key methodological components including the literature search and statistical analysis were missing or inadequate in most of included NMAs, such as only 22.50% of NMAs reported search strategy, 6.56% assessed the heterogeneity in NMAs. Methodological quality and reporting of statistical analysis did not substantially differ by selected general characteristics of NMAs.

NMAs could provide useful evidence on relative effectiveness of different interventions for decision-making when there are no or insufficient direct comparison trials^11^. Methodological quality of NMAs is a crucial point for health care decision-makers and researchers. We assessed the methodological quality of NMAs in the field of cancer based on modified AMSTAR checklist. Some methodological flaws were identified, especially regarding to literature search (Item 3), assessment of scientific quality (Item 7) and scientific quality used appropriately in formulating conclusions (Item 8), the methods used to combine the findings of studies (Item 9), and assessment of publication bias (Item 10).

NMAs aimed to rank the benefits (or harms) of interventions, based on all available RCTs. Thus, the identification of all relevant data is critical^7^. Most of the included NMAs (80.39%, 82/102) did not report database search strategy. For those that reported search strategy, 26.96% only searched previous published meta-analyses. It is important to search, track, and include previous systematic reviews and meta-analyses in conducting NMAs^22^. PubMed/MEDLINE was the most commonly used databases and the most common combination of databases was PubMed/MEDLINE and EMBASE. The majority of NMAs did not search Chinese databases. Cohen *et al.*’ study showed that searching Chinese databases might lead to the identification of a large amount of additional clinical evidence, and suggested that Chinese biomedical databases should be searched when performing systematic reviews^23^.

The assessment of scientific quality of individual studies could affect findings of NMAs^24^. However, 31.37% of the included NMAs did not report methods for assessing the risk of bias of individual studies in methods sections. And 69.61% did not consider the scientific quality of the included studies in formulating conclusions. Although reporting bias could have a substantial effect on the conclusions of a NMA^12^, most of the included NMAs (84.31%) did not report a method to assess publication bias.

The complex nature of NMA mainly reflected in the diversification of interventions and complex statistical analysis process. Homogeneity and consistency assumptions underlie NMA^25^. Although assessment of heterogeneity in traditional meta-analyses was common, only 4 NMAs (3.92%) assessed the heterogeneity in the entire network by heterogeneity variance parameter (Tau^2^). Eleven (10.78%) explicitly reported the methods of assessment of similarity. For those with Bayesian framework, 17 (27.87%) assessed the inconsistency between direct comparisons and indirect comparisons. GRADE tool was proposed to assess the quality of evidence from NMAs in 2014^26^. However, it still was rarely used to assess quality of evidence in NMAs related to cancer.

To the best of our knowledge, this is the first review to comprehensively assess the methodological quality using a modified AMSTAR checklist, and simultaneously assess the quality of reporting of literature search and statistical analysis methods. Two recent reviews that also focused on the methodological problems of published network meta analyses^12^,^15^ covered a wide range of medical areas and some details of reporting of literature search and statistical analysis were missing. Bafeta A *et al.*^12^ included 121 NMAs to examine the methodological reporting of NMAs, the results showed that 73% did not report the electronic search strategy for each database compared with 77.5% in our study. Most of NMAs did not assess quality of evidence using GRADE tool (3% vs. 4.92%). The results of methodological reporting were similar to our study. Chambers J *et al.*^15^ also showed that there were similar methodological quality problems in their included NMAs. However, AMSTAR checklist has not been used to systematically assess the methodological quality of NMAs. Furthermore, we explored the potential factors influencing methodological quality and statistical reporting according to general characteristics of the included NMAs. There were no substantial differences by selected general characteristics of NMAs.

Our study also have some limitations. There was no standard tool to assess the methodological quality of NMAs. We slightly modified three of the 11 AMSTAR items (Item 1, Item 5, and Item 9) to assess the methodological quality of NMAs. However, there are still some problems or uncertain issues, such as the difficulty in defining type of interventions and type of comparisons for inclusion in NMAs, how to draw geometry of the network, how to handle multi group trials, how to decide whether the assessment of similarity and consistency was appropriate, and whether statistical analysis methods were appropriate for NMAs. The complex nature of statistical analysis of NMAs raised the necessity to develop a guideline about the reporting of statistical analysis of NMAs. As with other methodological studies, assessing methodological quality and reporting quality from published reports alone could be misleading. The study authors may have used adequate methods but omitted important details from published reports^12^, or published reports were sufficient referring to relevant reporting guidelines but not rigorous during the conduct process. For example, while we distinguished whether study selection and data extraction were performed by least two independent reviewers, we did not know whether the processes were really performed by two independent reviewers. Finally, we did not identify any eligible NMAs related to diagnostic test accuracy and animal study. We also did not include reviews based on individual patient data (IPD) due to the differences of method and statistical analysis processes between IPD and aggregated data.

Overall, the methodological quality of NMAs in the field of cancer was generally acceptable. However, some methodological flaws have been identified in published NMAs, especially regarding to literature search, assessment of scientific quality and scientific quality used appropriately in formulating conclusions, the methods used to combine findings of studies, and assessment of publication bias. Methodological quality and statistical reporting did not substantially differ by general characteristics.

## Methods

### Search strategy

PubMed, EMBASE, Web of Science, Science Citation Index Expanded, Social Sciences Citation Index, The Cochrane Library, Cochrane Database of Systematic Reviews, Database of Abstracts of Reviews of Effects, Health Technology Assessment Database, NHS Economic Evaluation Database, Chinese Biomedical Literature Database (CBM), and China National Knowledge Infrastructure (CNKI) were searched from inception to February 26th, 2014. The search strategy was recently reported in a published paper^7^. All searches were updated on 9th July, 2015.

### Eligibility criteria

We included any NMAs in the field of cancer in the English and Chinese languages, regardless of interventions. NMAs were defined as meta-analyses that used network meta-analytic methods to analyze, simultaneously, three or more different interventions^7^, adjusted indirect comparisons were also included. If the same NMA had duplicate publications, the latest was included. We excluded methodological articles, conference abstracts, letters, editorials, correspondences, cost-effectiveness reviews, and reviews based on individual patient data.

### Study selection

Literature search records were imported into ENDNOTE X6 literature management software. Two independent reviewers (LG, LL) examined the title and abstract of retrieved studies to identify potentially relevant studies according to the eligibility criteria. Then, full-text versions of all potentially relevant studies were obtained. Excluded trials and the reasons for their exclusion were listed, conflicts were resolved by a third reviewer (J-HT, or K-HY).

### Data extraction and management

A standard data abstraction form was created using Microsoft Excel 2013 (Microsoft Corp, Redmond, WA, www.microsoft.com) to collect data of interest. A pilot-test was performed for literature selection and data extraction, and a “cheat sheet” with detailed definitions and examples were developed to ensure high inter-rater reliability among the reviewers.

### General characteristics

The following general characteristics were collected by one reviewer (LG): first author, year of publication, country of corresponding author, journal name, publishing period (time from received to accepted), funding source (industry-supported, non-industry-supported, unfunded or not report), number of author, language of publication (English or Chinese), number and type of included original studies, sample size of included original studies, number of study arm, type of outcome (dichotomous, continue, or survival time), categories of disease, and number of interventions included in the network. We categorised journal types into Science Citation Index (SCI) or non-SCI; we also identified journals with high impact factors (IF ≥ 5.000, as reported on Journal Citation Reports 2014)^27^ or low impact factors (IF < 5.000). We also categorised NMAs into older studies or recent studies based on the median division of number of included NMAs.

### Reporting of literature search

One reviewer (XQ) extracted following information regarding reporting of literature search: number of databases searched (Chinese, English, or both), name of databases searched, whether the search strategy was provided, whether the previous systematic reviews/meta-analyses were searched, name and number of other sources searched (e.g., reference lists checking, clinical trial registration platform, conference abstracts or web sites, Google engine).

### Reporting of statistical analysis processes

We assessed the reporting and quality of statistical analysis processes in the methods sections of each NMA report according to the Preferred Reporting Items for Systematic Reviews and Meta-analyses (PRISMA) extension statement for NMAs^28^. The following questions were designed according to the statistical analysis section of PRISMA extension statement, and were extracted by two independent reviewers (LG, JZ), and conflicts were resolved by a third reviewer (J-HT, or K-HY):

- Was traditional meta-analysis conducted?
- Were summary measures reported?State the principal summary measures (e.g., risk ratio, odd ratio, mean difference, hazard ratio). Also describe the use of additional summary measures assessed, such as treatment rankings (e.g., treatment rankings, best, or surface under the cumulative ranking curve (SUCRA) values), shape and scale parameters for survival data^29^.
- Planned methods of analysis, this should include: Was a Bayesian or a frequentist framework used? What was the model used? (random-effects model, fixed-effect model, others, or not report). What was the method used to undertake the indirect comparisons^30^?Was the model code presented or source cited in Bayesian analyses? (not provided, manuscript, online supplement, external web site, reference, or others). Was the model fit assessed? (e.g., residual deviance^31^, deviance information criterion^31^, other, not reported). Was handling of multigroup trials reported? Was selection of prior distributions in Bayesian analyses described? Was the convergence in Bayesian analyses assessed^32^? Was the heterogeneity in traditional meta-analysis assessed and how to handle the heterogeneity^33^? Was the heterogeneity in the entire network of NMA assessed and how to handle the heterogeneity^34^? Was the transitivity/similarity in NMA assessed^35^?
- Were the inconsistency assessed and how to handle the inconsistency^36^?
- Was the publication or reporting bias assessed^37^?
- Additional analyses included: Was a sensitivity analysis performed? (e.g., excluding studies, alternative prior distributions for Bayesian analyses, alternative formulations of the treatment network). Was subgroup analysis or meta-regression performed?
- Was the Grading of Recommendations Assessment, Development and Evaluation (GRADE) tool used to assess quality of evidence^26^?

### Methodological quality assessment

There were no consensuses to assess the methodological quality of NMAs. We assessed the methodological quality of included NMAs using a modified AMSTAR checklist. This checklist included 11 items, with possible responses of “Yes” (item/question fully addressed), “No” (item/question not addressed), “Cannot answer” (not enough information to answer the question), and “Not applicable”. Two reviewers (XQ, G-QP) independently extracted data, and conflicts were resolved by a third reviewer (LG, or J-HT). The total score using AMSTAR was obtained by summing one point for each “yes” and no points for any other responses (“no”, “Cannot answer” and “Not applicable”), ranging from 0 to 11. In our study, three of the 11 items were slightly modified as follows (Appendix 3):

- “Was an ‘a priori’ design provided?” was amended to “Was the research question (i.e., research purpose, inclusion and exclusion criteria) clarified?”The reason for this modification was that only a small minority of published non-Cochrane reviews reported a protocol^38^. Where a protocol providing this information was available, the answer to this question would be “Yes”. Where no protocol was available but detailed information about research purpose and inclusion and exclusion criteria (patients, interventions, comparators, outcome, and study design) were supplied, we also considered answer this question “Yes”.
- “Was a list of studies (included and excluded) provided?” was amended to “Were a list of included studies and flow diagram provided?”The reason for this modification was that most of published systematic reviews did not provide a list of excluded studies. Where a list of included studies and flow diagram of literature selection were provided (as references, electronic link, or supplement), we considered answer this question “Yes”.
- Were the methods used to combine the findings of studies appropriate?For pairwise meta-analysis, we scored “Yes” if they mentioned or described heterogeneity and reported how to handle heterogeneity. For NMA, the following factors should be taken into consideration except heterogeneity, but not be limited to: summary measures, model used, model fit, prior distributions (Bayesian analysis), convergence (Bayesian analysis), and inconsistency.

### Statistical analysis

Quantitative data were summarised by medians and interquartile range (IQR), and categorical data summarised by numbers and percentages. The association between methodological quality and following characteristic variables was explored using the Mann-Whitney U test and Kruskai-Wallis test: journal impact factor, year of publication, funding source, country of corresponding author, type of NMAs, and categories of disease. Moreover, the subgroup analyses for statistical reporting were performed according to journal impact factor (high vs. low impact factors) and year of publication (older vs. recent studies). Proportion results were analysed by Chi-square test using STATA version 12.0^39^. All tests were two sided, and P ≤ 0.05 was considered statistically significant.

## Additional Information

**How to cite this article**: Ge, L. *et al.* Epidemiology Characteristics, Methodological Assessment and Reporting of Statistical Analysis of Network Meta-Analyses in the Field of Cancer. *Sci. Rep.* **6**, 37208; doi: 10.1038/srep37208 (2016).

**Publisher’s note**: Springer Nature remains neutral with regard to jurisdictional claims in published maps and institutional affiliations.

## Supplementary Material

Supplementary Information

Supplementary Dataset

Supplementary Information

## Figures and Tables

**Table 1:** Characteristics of the included NMA.

	Category	Frequency	Proportion [%(95% CI)]
Language (n = 102)	English	92	90.20 (82.70–94.60)
	Chinese	10	9.80 (5.40–17.30)
Journal quality (n = 102)	SCI	87	85.30 (77.00–90.90)
	Non-SCI	15	14.70 (9.10–23.00)
	CSCD	3	2.90 (1.00–8.70)
	Non-CSCD	7	6.90 (3.30–13.70)
Impact factor (n = 87)	Median (IQR)	3.94 (2.721–5.635)	
	0.0–2.0	6	6.90 (3.10–14.50)
	2.0–5.0	49	56.30 (45.80–66.30)
	5.0–10.0	22	25.30 (17.30–35.40)
	≥10.0	10	11.50 (6.30–20.10)
Publishing period (n = 56)	Median (IQR)	101 (47–187) day	
	1–30 days	12	21.40 (12.60–34.10)
	30–60 days	10	17.90 (9.90–30.10)
	60–90 days	4	7.10 (2.70–17.50)
	90–150 days	12	21.40 (12.60–34.10)
	≥150 days	19	33.90 (22.80–47.20)
Funding source (n = 60)	Unfunded (n = 14)	14	23.30 (14.30–35.60)
	Industry-supported (n = 20)	20	33.30 (22.60–46.10)
	Government-supported (n = 23)	23	38.30 (27.00–51.10)
	Industry + Government (n = 3)	3	5.00 (1.60–14.40)
Number of author	Median (IQR)	6 (5–8)	
Type of included study (n = 102)	RCT	100	98.00 (92.50–99.50)
	Meta-analysis	2	2.00 (0.50–7.50)
Number of included trial	Median (IQR)	12 (7–23)	
Intervention	Median (IQR)	5 (3–9)	
Sample size (n = 102)	Median (IQR)	3605 (1950–7564)	
	Not reported	7	6.90 (3.30–13.70)
Outcome (n = 102)	Median (IQR)	3 (2–5)	
	Dichotomous	73	71.60 (62.10–79.50)
	Continue	5	4.90 (2.10–11.20)
	Survival time	79	77.50 (68.30–84.50)
Number of study arm (n = 102)	Two arms	50	49.00 (39.50–58.60)
	Three arms	7	6.90 (3.30–13.70)
	Two + Three arms/more	36	35.30 (26.70–45.00)

Note: CI, confidence interval; NMA, network meta-analysis; SCI, Science Citation Index; CSCD, Chinese Science Citation Database; IQR, interquartile range.

**Table 2:** Reporting information of literature search.

Category		Frequency	Proportion **[%(95%** CI)]
Reported database searched (n = 102)	Yes	89	87.30 (79.30–92.50)
Language of database searched (n = 89)*	English	88	98.90 (92.50–99.80)
	Chinese	10	11.20 (6.20–19.60)
	Chinese + English	9	10.10 (5.30–18.30)
Number of Chinese database searched	Median (IQR)	5(3–6)	
Number of English database searched	Median (IQR)	3(3–4)	
Number of search strategy	Median (IQR)	2(1–3)	
Reported search strategy (n = 89)	Yes	20	22.50 (15.00–32.30)
Presented search strategy (n = 20)	Manuscript	9	45.00 (25.30–66.40)
	Supplement	5	25.00 (10.80–47.80)
	Online supplement	6	30.00 (14.10–52.70)
Searched previous published meta analysis (n = 89)	Yes	24	27.00 (18.80–37.10)
Supplemental literature search (n = 89)	Yes	75	84.30 (75.20–90.50)
Supplemental literature search method (n = 75)	Reference lists checking	42	56.00 (44.70–66.80)
	Clinical trial registration platform	27	36.00 (26.00–47.40)
	Conference abstracts or Web sites	43	57.30 (46.00–68.00)
	Google engine	17	22.70 (14.60–33.50)

^*^13 studies did not search databases. CI, confidence interval; IQR, interquartile range.

**Table 3:** Information of databases searched (n = 89).

	Category	Frequency	Proportion **[%(95%** CI)]
Name of database	PubMed/MEDLINE	84	94.40 (87.20–97.60)
	EMBASE	69	77.50 (67.70–85.00)
	The Cochrane Library	71	79.80 (70.20–86.90)
	ISI Web of Knowledge	17	19.10 (12.20–28.60)
	CNKI	9	10.10 (5.30–18.30)
	CBM	9	10.10 (5.30–18.30)
	Wanfang	7	7.90 (3.80–15.60)
	VIP	8	9.00 (4.60–17.00)
	CSCD	7	7.90 (3.80–15.60)
	Others	35	39.30 (29.80–49.80)
Common combination of database	PubMed/MEDLINE + EMBASE	65	73.00 (62.90–81.20)
	PubMed/MEDLINE + Cochrane	69	77.50 (67.70–85.00)
	EMBASE + Cochrane	60	67.40 (57.00–76.30)
	PubMed/MEDLINE + EMBASE + Cochrane	56	62.90 (52.50–72.30)
	PubMed/MEDLINE + ISI Web of Knowledge	17	19.10 (12.20–28.60)
	PubMed/MEDLINE + EMBASE + Cochrane + ISI Web of Knowledge	14	15.70 (9.50–24.80)
	PubMed/MEDLINE + EMBASE + CBM	8	9.00 (4.60–17.00)
	PubMed/MEDLINE + CNKI	7	7.90 (3.80–15.60)

**Table 4:** Statistical Reporting in Bayesian analysis [n/N(%)].

Items	All studies (n = 61)	Journal impact factor*			Year of publication		
		Low (<5.00)(n = 26)	High (≥5.00)(n = 26)	P-value	Older studies(~2013) (n = 29)	Recent studies(2014~2015) (n = 32)	P-value
Was traditional meta-analysis conducted? (Yes)	42/61(68.85)	15/26(57.69)	20/26(76.92)	0.143	18/29(62.07)	24/32(93.10)	0.280
Were summary measures reported? (Yes)	57/61(93.44)	24/26(92.31)	25/26(96.15)	0.556	27/29(93.10)	30/32(93.75)	0.920
Planned methods of analysis:							
What was the model used?							
Fixed-effect	7/46(15.22)	4/20(20.00)	2/24(8.33)	0.267	6/26(23.08)	1/20(5.00)	0.094
Random-effects	11/46(23.91)	4/20(20.00)	6/24(25.00)	0.697	6/26(23.08)	5/20(25.00)	0.881
Fixed- and random-effects	18/46(39.13)	12/20(60.00)	6/24(25.00)	**0.020**	7/26(26.92)	11/20(55.00)	0.056
Other models	10/46(21.74)	0/20(0.00)	10/24(41.67)	**0.001**	7/26(26.92)	3/20(15.00)	0.336
Nor reported	15/61(24.59)	6/26(23.08)	2/26(7.69)	0.128	3/29(10.34)	12/32(37.50)	**0.015**
How to present the model code?							
Supplement	5/21(23.81)	2/6(33.33)	3/15(20.00)	0.527	2/14(14.29)	3/7(42.86)	0.157
Reference	16/21(76.19)	4/6(66.67)	12/15(80.00)	0.527	12/14(85.71)	4/7(57.14)	0.157
Not provided	40/61(65.57)	20/26(76.92)	11/26(42.31)	**0.012**	15/29(51.72)	25/32(78.13)	**0.032**
How to assess the model fit?							
Residual deviance	2/24(8.33)	1/14(7.14)	1/10(10.00)	0.807	1/9(11.11)	1/15(6.67)	0.709
Deviance information criterion	15/24(62.50)	8/14(57.14)	7/10(70.00)	0.530	6/9(66.67)	9/15(60.00)	0.749
Residual deviance+Deviance information criterion	7/24(29.17)	5/14(35.71)	2/10(20.00)	0.414	2/9(22.22)	5/15(33.33)	0.570
Not reported	37/61(60.66)	12/26(46.15)	16/26(61.54)	0.270	20/29(68.97)	17/32(53.13)	0.210
Was handling of multigroup trials reported? (Yes)	5/61(8.20)	1/26(3.85)	3/26(11.54)	0.303	3/29(10.34)	2/32(6.25)	0.564
Were the prior distributions reported?							
Noninformative prior	18/31(58.06)	9/13(69.23)	10/17(58.82)	0.564	8/16(50.00)	10/15(66.67)	0.355
Informative prior	3/31(9.68)	1/13(7.69)	2/17(11.76)	0.717	1/16(6.25)	2/15(13.33)	0.512
Vague prior	10/31(32.26)	4/13(30.77)	6/17(35.29)	0.798	7/16(43.75)	3/15(20.00)	0.164
Not specified	30/61(49.18)	13/26(50.00)	9/26(34.62)	0.266	13/29(44.83)	17/32(53.13)	0.521
Sensitivity analysis based on priors	4/61(6.56)	2/26(7.69)	2/26(7.69)	1.000	2/29(6.90)	2/32(6.25)	0.920
Was the convergence assessed?							
Not reported	37/61(60.66)	15/26(57.69)	14/26(53.85)	0.782	17/29(58.62)	20/32(62.50)	0.759
Gelman Rubin statistic	21/24(87.50)	11/11(100.00)	10/12(83.33)	0.166	9/12(75.00)	12/12(100.00)	0.070
Visual plot inspection	13/24(54.17)	5/11(45.45)	7/12(58.33)	0.546	7/12(58.33)	6/12(50.00)	0.688
Observation of chain mix	2/24(8.33)	2/11(18.18)	0/12(0.00)	0.131	1/12(8.33)	1/12(8.33)	1.000
Was the heterogeneity in NMA assessed?							
Precision (Tau^2^)	4/61(6.56)	0/26(0.00)	4/26(15.38)	**0.039**	4/29(13.79)	0/32(0.00)	**0.031**
Not reported	57/61(93.44)	26/26(100.00)	22/26(84.62)	**0.039**	25/29(86.21)	32/32(100.00)	**0.031**
Was the similarity in NMA assessed?							
Not reported	53/61(86.89)	22/26(84.62)	22/26(84.62)	1.000	23/29(79.31)	30/32(93.75)	0.098
Comparing patients’ or trials’ characteristics	5/8(62.50)	2/4(50.00)	3/4(75.00)	0.495	4/6(66.67)	1/2(50.00)	0.693
Investigating potential effect modifying covariates	3/8(37.50)	2/4(50.00)	1/4(25.00)	0.495	2/6(33.33)	1/2(50.00)	0.693
Were the inconsistency assessed?							
Not reported	44/61(72.13)	20/26(76.92)	16/26(61.54)	0.234	20/29(68.97)	24/32(75.00)	0.603
Inconsistency/incoherence factors	4/17(23.53)	1/6(16.67)	2/10(20.00)	0.873	3/9(33.33)	1/8(12.50)	0.327
Hypothesis test	8/17(47.06)	2/6(33.33)	6/10(60.00)	0.317	4/9(44.44)	4/8(50.00)	0.824
Node-splitting analysis	5/17(29.41)	3/6(50.00)	2/10(20.00)	0.225	2/9(22.22)	3/8(37.50)	0.503
Was the publication or reporting bias assessed?							
Comparison-adjusted funnel plot	1/61(1.64)	0/26(0.00)	1/26(3.85)	0.317	0/29(0.00)	1/32(3.13)	0.341
Not reported	60/61(98.36)	26/26(100.00)	25/26(96.15)	0.317	29/29(100.00)	31/32(96.87)	0.341
Additional analyses:							
Was a sensitivity analysis performed? (Yes)	24/61(39.34)	10/26(38.46)	12/26(46.15)	0.578	12/29(41.38)	12/32(37.50)	0.759
Was subgroup analysis or meta-regression performed? (Yes)	12/61(19.67)	3/26(11.54)	7/26(26.92)	0.163	7/29(24.14)	5/32(15.63)	0.407
Was the GRADE used? (Yes)	3/61(4.92)	1/26(3.85)	2/26(7.69)	0.556	2/29(6.90)	1/32(3.13)	0.500

^*^9 studies published in journals with no associated impact factor.

**Table 5:** Statistical Reporting in adjusted indirect comparisons [n/N(%)].

Items	n/N	Journal impact factor (n = 37)*			Year of publication (n = 43)#		
		Low (<5.00)(n = 29)	High (≥5.00)(n = 8)	P-value	Older studies(~2013) (n = 31)	Recent studies(2014~2015) (n = 12)	P-value
Was traditional meta-analysis conducted? (Yes)	42/43(97.67)	28/29(96.55)	8/8(100.00)	0.599	30/31(96.77)	12/12(100.00)	0.534
Were summary measures reported? (Yes)	41/43(97.62)	26/29(89.66)	8/8(100.00)	0.976	29/31(93.55)	12/12(100.00)	0.373
Planned methods of analysis in adjusted indirect comparisons:							
What was the method used?							
Bucher	23/28(82.14)	17/20(85.00)	2/4(50.00)	0.140	15/20(75.00)	8/8(100.00)	0.240
Other methods	5/28(17.86)	3/20(15.00)	2/4(50.00)	0.123	5/20(25.00)	0/8(0.00)	0.125
Not reported	15/43(34.88)	9/29(31.03)	4/8(50.00)	0.326	11/31(35.48)	4/12(33.33)	0.896
Was the method presented or source cited?							
Manuscript	5/23(21.74)	1/16(6.25)	2/4(50.00)	**0.033**	4/20(20.00)	1/3(33.33)	0.610
Reference	18/23(78.26)	15/16(93.75)	2/4(50.00)	**0.033**	16/20(80.00)	2/3(66.67)	0.610
Not provided	20/43(46.51)	13/29(44.83)	4/8(50.00)	0.798	11/31(35.48)	9/12(75.00)	0.021
Was handling of multigroup trials reported? (Yes)	2/43(4.65)	0/29(0.00)	1/8(12.50)	0.057	2/31(6.45)	0/12(0.00)	0.373
Was the similarity assessed?	3/43(6.98)	3/29(10.34)	0/8(0.00)	0.349	3/31(9.68)	0/12(0.00)	0.269
Was the inconsistency assessed?							
Not reported	36/43(83.72)	25/29(86.21)	5/8(62.50)	0.150	26/31(83.87)	10/12(83.33)	0.970
Not application	4/7(57.14)	4/4(100.00)	0/3(0.00)	0.050	3/5(60.00)	1/2(50.00)	0.810
Hypothesis test	3/7(42.86)	0/4(0.00)	3/3(100.00)	0.050	2/5(40.00)	1/2(50.00)	0.810
Additional analyses:							
Was a sensitivity analysis performed?	5/43(11.63)	1/29(3.45)	3/8(37.50)	0.007	3/31(9.68)	2/12(16.67)	0.526
Was subgroup analysis or meta-regression performed?	9/43(20.93)	8/29(27.59)	1/8(12.50)	0.385	6/31(19.35)	3/12(25.00)	0.827

^*^6 studies published in journals with no associated impact factor.

^#^Based on the median division of number of included NMAs, December 31st 2013 is the cut-off point.

**Table 6:** Subgroup analyses of methodological quality assessment (n/%).

Items (Yes)	All NMAs (n = 102)	Journal impact factor*		Year of publication#		Funding source		Country of corresponding author		Type of NMAs	
		Low (<5.00) (n = 55) vs. High (≥5.00) (n = 32)	P-value	Older (n = 58) vs. Recent (n = 44)	P-value	Funding (n = 46) vs. Non-funding (n = 56)	P-value	China (n = 29) vs. Others (n = 73)	P-value	Bayesian NMAs (n = 61) vs. Adjusted indirect comparisons (n = 43)&	P-value
Was the research question (i.e., research purpose, inclusion and exclusion criteria) clarified?	62/60.78	31/56.36 vs. 20/62.50	0.568	33/56.90 vs. 29/65.91	0.358	26/56.52 vs. 36/64.29	0.426	20/68.97 vs. 42/57.53	0.288	38/62.30 vs. 25/58.14	0.671
Was there duplicate study selection and data extraction?	74/72.55	38/69.09 vs. 24/75.00	0.547	41/70.69 vs. 33/75.00	0.631	32/69.57 vs. 42/75.00	0.543	26/89.66 vs. 48/65.75	**0.015**	50/81.97 vs. 26/60.47	**0.015**
Was a comprehensive literature search performed?	58/56.86	25/45.45 vs. 25/78.13	**0.002**	31/53.45 vs. 27/61.36	0.426	26/56.52 vs. 32/57.14	0.950	16/55.17 vs. 42/57.53	0.829	43/70.49 vs. 17/39.53	**0.002**
Was the status of publication (i.e. grey literature) used as an inclusion criterion?	81/79.41	43/78.18 vs. 26/81.25	0.728	46/79.31 vs. 35/79.55	0.977	35/76.09 vs. 46/82.14	0.454	26/89.66 vs. 55/75.34	0.109	53/86.89 vs. 29/67.44	**0.017**
Was a list of included studies provided?	94/92.16	50/90.91 vs. 29/90.63	0.965	53/91.38 vs. 41/93.18	0.739	44/95.65 vs. 50/89.29	0.236	28/96.55 vs. 66/90.41	0.300	56/91.80 vs. 40/93.02	0.819
Were the characteristics of the included studies provided?	100/98.04	54/98.18 vs. 31/96.88	0.715	58/100.00 vs. 42/95.45	0.103	45/97.83 vs. 55/98.21	0.889	29/100.00 vs. 71/97.26	0.370	59/96.72 vs. 43/100.00	0.233
Was the scientific quality of the included studies assessed and documented?	70/68.63	34/61.82 vs. 25/78.13	0.098	32/55.17 vs. 38/86.36	**0.001**	29/63.04 vs. 41/73.21	0.273	25/86.21 vs. 45/61.64	**0.016**	48/78.69 vs. 24/55.81	**0.013**
Was the scientific quality of the included studies used appropriately in formulating conclusions?	31/30.39	11/20.00 vs. 8/25.00	0.584	12/20.69 vs. 19/43.18	**0.015**	9/19.57 vs. 22/39.29	**0.032**	20/68.97 vs. 11/15.07	**0.000**	21/34.43 vs. 10/23.26	**0.222**
Were the methods used to combine the findings of studies appropriate?	66/64.71	32/58.18 vs. 26/81.25	**0.019**	37/63.79 vs. 29/65.91	0.826	28/60.87 vs. 38/67.86	0.465	18/62.07 vs. 48/65.75	0.727	39/63.93 vs. 29/67.44	0.713
Was the likelihood of publication bias assessed?	16/15.69	3/5.45 vs. 8/25.00	**0.017**	10/17.24 vs. 6/13.64	0.622	5/10.87 vs. 11/19.64	0.228	6/20.69 vs. 10/13.70	0.383	8/13.11 vs. 9/20.93	0.291
Was the conflict of interest stated?	62/60.78	38/69.09 vs. 22/68.75	0.974	37/63.79 vs. 25/56.82	0.477	29/63.04 vs. 33/58.93	0.673	11/37.93 vs. 51/69.86	**0.001**	35/57.38 vs. 27/62.79	0.581

^*^6 studies published in journals with no associated impact factor.

^#^Based on the median division of number of included NMAs, December 31st 2013 is the cut-off point.

^&^2 adjusted indirect comparisons also were conducted using Bayesian framework.

**Table 7:** The association of total AMSTAR-score and selected general characteristics [median (IQR)].

Category	Overall score	AMSTAR-score	Statistic	P-value
		8.00 (6.00, 8.25)		
Year of publication			1.194	0.232
	2006~2013 (n = 58)	7.00(5.00, 9.00)		
	2014~2015 (n = 44)	8.00(6.25, 8.00)		
Country of corresponding author I			2.557	0.465
	East Asia (n = 34)	8.00(7.00, 9.00)		
	Europe (n = 47)	7.00(5.00, 8.00)		
	North America (n = 18)	7.00(5.00, 8.25)		
	Others (n = 3)	7.00(6.00, 7.00)		
Country of corresponding author II			2.272	0.023
	China (n = 29)	8.00(7.00, 9.00)		
	Others (n = 73)	7.00(5.00, 8.00)		
Impact factors of SCI			5.503	0.239
	Not SCI (n = 15)	8.00(6.00, 8.00)		
	0.0–2.0 (n = 6)	5.00(2.00, 9.00)		
	2.0–5.0 (n = 49)	7.00(5.00, 8.00)		
	5.0–10.0 (n = 22)	8.00(6.00, 9.00)		
	≥10.0 (n = 10)	7.00(7.00, 9.00)		
Funding source			1.512	0.469
	Not report (n = 42)	8.00(6.00, 9.00)		
	Unfunded (n = 14)	7.50(7.00, 8.00)		
	Funding-supported (n = 46)	7.00(5.00, 8.25)		
Method			1.601	0.109
	Indirect comparison (n = 41)	7.00(3.50, 8.00)		
	Bayes (n = 61)	8.00(6.00, 9.00)		
Categories of disease			5.649	0.342
	Non-small cell lung cancer (n = 19)	8.00(7.00, 9.00)		
	Breast cancer (n = 12)	7.00(4.25, 8.00)		
	Renal cell carcinoma (n = 7)	6.00(5.00, 8.00)		
	Colorectal cancer (n = 6)	7.50(6.00, 10.25)		
	Gastric cancer (n = 6)	8.00(7.00, 9.00)		
	Others (n = 52)	7.50(5.25, 8.75)		
