# Supplementary material for: Epidemiology Characteristics, Methodological Assessment and Reporting of Statistical Analysis of Network Meta-Analyses in the Field of Cancer
Source: Sci Rep. 2016 Nov 16;6:37208. doi: 10.1038/srep37208 (PMC5111127; doi:10.1038/srep37208)
Supplement: Supplementary Information [file srep37208-s1.pdf]

Epidemiology Characteristics, Methodological Assessment and Reporting of Statistical Analysis of Network Meta-Analyses in the Field of Cancer

Long Ge, Jin-hui Tian, Xiu-xia Li, Fujian Song, Lun Li, Jun Zhang, Ge Li, Gai-qin Pei, Xia Qiu, Ke-hu Yang

Appendix 1--A list of included network meta-analyses[1-102]

1 Kumagai, K. *et al.* Meta-analysis of postoperative morbidity and perioperative mortality in patients receiving neoadjuvant chemotherapy or chemoradiotherapy for resectable oesophageal and gastro-oesophageal junctional cancers. *Br J Surg* **101**, 321-338, doi:10.1002/bjs.9418 (2014).

2 Bachelot, T. *et al.* Comparative efficacy of everolimus plus exemestane versus fulvestrant for hormone-receptor-positive advanced breast cancer following progression/recurrence after endocrine therapy: a network meta-analysis. *Breast Cancer Res Treat* **143**, 125-133, doi:10.1007/s10549-013-2778-5 (2014).

3 Palmieri, C., Fullarton, J. R. & Brown, J. Comparative efficacy of bisphosphonates in metastatic breast and prostate cancer and multiple myeloma: a mixed-treatment meta-analysis. *Clin Cancer Res* **19**, 6863-6872, doi:10.1158/1078-0432.CCR-13-2275 (2013).

4 Signorovitch, J., Ayyagari, R., Reichmann, W. M., Wu, E. Q. & Chen, L. Major molecular response during the first year of dasatinib, imatinib or nilotinib treatment for newly diagnosed chronic myeloid leukemia: a network meta-analysis. *Cancer Treat Rev* **40**, 285-292, doi:10.1016/j.ctrv.2013.09.004 (2014).

5 Stern, C. Flexible sigmoidoscopy versus fecal occult blood testing for colorectal cancer screening in asymptomatic individuals. *Clinical Journal of Oncology Nursing* **18**, 471-472 (2014).

6 Dranitsaris, G., Schmitz, S. & Broom, R. J. Small molecule targeted therapies for the second-line treatment for metastatic renal cell carcinoma: a systematic review and indirect comparison of safety and efficacy. *J Cancer Res Clin Oncol* **139**, 1917-1926, doi:10.1007/s00432-013-1510-5 (2013).

7 Liao, W.-C. *et al.* Adjuvant treatments for resected pancreatic adenocarcinoma: a systematic review and network meta-analysis. *The Lancet Oncology* **14**, 1095-1103, doi:10.1016/s1470-2045(13)70388-7 (2013).

8 Pechlivanoglou, P., Le, H. H., Daenen, S., Snowden, J. A. & Postma, M. J. Mixed treatment comparison of prophylaxis against invasive fungal infections in neutropenic patients receiving therapy for haematological malignancies: a systematic review. *J Antimicrob Chemother* **69**, 1-11, doi:10.1093/jac/dkt329 (2014).

9 Skoetz, N. *et al.* Effect of initial treatment strategy on survival of patients with advanced-stage Hodgkin's lymphoma: a systematic review and network meta-analysis. *The Lancet Oncology* **14**, 943-952, doi:10.1016/s1470-2045(13)70341-3 (2013).

10 Bakalos, G. *et al.* Assessing the relative effectiveness and tolerability of treatments in small cell lung cancer: a network meta-analysis. *Cancer Epidemiol* **37**, 675-682, doi:10.1016/j.canep.2013.06.008 (2013).

11 Robertson, C. *et al.* Relative effectiveness of robot-assisted and standard laparoscopic prostatectomy as alternatives to open radical prostatectomy for treatment of localised prostate cancer: a systematic review and mixed treatment comparison meta-analysis. *BJU Int* **112**, 798-812, doi:10.1111/bju.12247 (2013).

12 Brown, T. *et al.* Clinical effectiveness and cost-effectiveness of first-line chemotherapy for adult patients with locally advanced or metastatic non-small cell lung cancer: a systematic review and economic evaluation. *Health Technol Assess* **17**, 1-278, doi:10.3310/hta17310 (2013).

13 Fang, Y. *et al.* Radioiodine therapy for patients with differentiated thyroid cancer after thyroidectomy: direct comparison and network meta-analyses. *J Endocrinol Invest* **36**, 896-902, doi:10.3275/8998 (2013).

14 Cope, S., Ouwens, M. J., Jansen, J. P. & Schmid, P. Progression-free survival with fulvestrant 500 mg and alternative endocrine therapies as second-line treatment for advanced breast

cancer: a network meta-analysis with parametric survival models. *Value Health* **16**, 403-417, doi:10.1016/j.jval.2012.10.019 (2013).

15 Das, R., Cope, S., Ouwens, M., Turner, P. & Howlett, M. Economic evaluation of fulvestrant 500 mg versus generic nonsteroidal aromatase inhibitors in patients with advanced breast cancer in the United Kingdom. *Clin Ther* **35**, 246-260 e245, doi:10.1016/j.clinthera.2013.01.011 (2013).

16 Mealing, S. *et al.* The relative efficacy of imatinib, dasatinib and nilotinib for newly diagnosed chronic myeloid leukemia: a systematic review and network meta-analysis. *Exp Hematol Oncol* **2**, 5, doi:10.1186/2162-3619-2-5 (2013).

17 Zou, Y. *et al.* Lenalidomide versus thalidomide based regimens as first-line therapy for patients with multiple myeloma. *Leuk Lymphoma* **54**, 2219-2225, doi:10.3109/10428194.2013.774393 (2013).

18 Larkin, J. *et al.* Second-line treatments for the management of advanced renal cell carcinoma: systematic review and meta-analysis. *Expert Opinion on Pharmacotherapy* **14**, 27-39 (2012).

19 Dequen, P. *et al.* Systematic review and network meta-analysis of overall survival comparing 3 mg/kg ipilimumab with alternative therapies in the management of pretreated patients with unresectable stage III or IV melanoma. *Oncologist* **17**, 1376-1385, doi:10.1634/theoncologist.2011-0427 (2012).

20 Ford, J. A. *et al.* Denosumab for treatment of bone metastases secondary to solid tumours: systematic review and network meta-analysis. *Eur J Cancer* **49**, 416-430, doi:10.1016/j.ejca.2012.07.016 (2013).

21 Riemsma, R. *et al.* Systematic review of lapatinib in combination with letrozole compared with other first-line treatments for hormone receptor positive(HR+) and HER2+ advanced or metastatic breast cancer(MBC). *Curr Med Res Opin* **28**, 1263-1279, doi:10.1185/03007995.2012.707643 (2012).

22 Terasawa, T., Trikalinos, N. A., Djulbegovic, B. & Trikalinos, T. A. Comparative efficacy of first-line therapies for advanced-stage chronic lymphocytic leukemia: a multiple-treatment meta-analysis. *Cancer Treat Rev* **39**, 340-349, doi:10.1016/j.ctrv.2012.05.007 (2013).

23 Lee, E. K. *et al.* Clinical and cost effectiveness of bevacizumab + FOLFIRI combination versus FOLFIRI alone as first-line treatment of metastatic colorectal cancer in South Korea. *Clin Ther* **34**, 1408-1419, doi:10.1016/j.clinthera.2012.05.001 (2012).

24 Mhaskar, R. et al. Bisphosphonates in multiple myeloma: a network meta-analysis. Cochrane Database Syst Rev**;(5)**:CD003188 (2012).

25 Wang, S. Y. *et al.* Network meta-analysis of margin threshold for women with ductal carcinoma in situ. *J Natl Cancer Inst* **104**, 507-516, doi:10.1093/jnci/djs142 (2012).

26 Qi, W. X. *et al.* Erlotinib and pemetrexed as maintenance therapy for advanced non-small-cell lung cancer: a systematic review and indirect comparison. *Curr Med Res Opin* **28**, 643-650, doi:10.1185/03007995.2012.675880 (2012).

27 Cheng, M. M., Goulart, B., Veenstra, D. L., Blough, D. K. & Devine, E. B. A network meta-analysis of therapies for previously untreated chronic lymphocytic leukemia. *Cancer Treat Rev* **38**, 1004-1011, doi:10.1016/j.ctrv.2012.02.006 (2012).

28 Fleeman, N. *et al.* Lapatinib and trastuzumab in combination with an aromatase inhibitor for the first-line treatment of metastatic hormone receptor-positive breast cancer which over-expresses human epidermal growth factor 2 (HER2): a systematic review and economic analysis. *Health Technology Assessment* **15**, 1-100 (2011).

29 Levy, A. R. *et al.* Indirect comparison of the efficacy of cetuximab and cisplatin in squamous cell carcinoma of the head and neck. *Curr Med Res Opin* **27**, 2253-2259, doi:10.1185/03007995.2011.633989 (2011).

30 Nuijten, M. J. *et al.* An indirect comparison of the efficacy of bevacizumab plus carboplatin and paclitaxel versus pemetrexed with cisplatin in patients with advanced or recurrent non-squamous adenocarcinoma non-small cell lung cancer. *Curr Med Res Opin* **27**, 2193-2201, doi:10.1185/03007995.2011.626019 (2011).

31 Mickisch, G. H. *et al.* Indirect treatment comparison of bevacizumab + interferon-alpha-2a vs tyrosine kinase inhibitors in first-line metastatic renal cell carcinoma therapy. *Clinicoecon Outcomes Res* **3**, 19-27, doi:10.2147/CEOR.S16118 (2011).

32 Gines, J. *et al.* Efficacy of taxanes as adjuvant treatment of breast cancer: a review and meta-analysis of randomised clinical trials. *Clin Transl Oncol* **13**, 485-498,

doi:10.1007/s12094-011-0686-x (2011).
33 Leung, H. W. & Chan, A. L. Multikinase inhibitors in metastatic renal cell carcinoma: indirect comparison meta-analysis. *Clin Ther* **33**, 708-716, doi:10.1016/j.clinthera.2011.05.003 (2011).
34 Sjoquist, K. M. *et al.* Survival after neoadjuvant chemotherapy or chemoradiotherapy for resectable oesophageal carcinoma: an updated meta-analysis. *Lancet Oncology* **12**, 681-692 (2011).
35 Ziogas, D. C., Voulgarelis, M. & Zintzaras, E. A network meta-analysis of randomized controlled trials of induction treatments in acute myeloid leukemia in the elderly. *Clin Ther* **33**, 254-279, doi:10.1016/j.clinthera.2011.04.004 (2011).
36 Chang, J. W. *et al.* An indirect comparison of bevacizumab plus cisplatin-gemcitabine and cisplatin plus pemetrexed treatment for patients with advanced first-line non-squamous non-small cell lung cancer in East Asia. *Asia Pac J Clin Oncol* **7 Suppl 2**, 13-21, doi:10.1111/j.1743-7563.2011.01398.x (2011).
37 Blanchard, P. *et al.* Mixed treatment comparison meta-analysis of altered fractionated radiotherapy and chemotherapy in head and neck cancer. *J Clin Epidemiol* **64**, 985-992, doi:10.1016/j.jclinepi.2010.10.016 (2011).
38 Bangalore, S. *et al.* Antihypertensive drugs and risk of cancer: network meta-analyses and trial sequential analyses of 324,168 participants from randomised trials. *Lancet Oncology* **12**, 65-82 (2011).
39 Kumar, A., Hozo, I., Wheatley, K. & Djulbegovic, B. Thalidomide versus bortezomib based regimens as first-line therapy for patients with multiple myeloma: a systematic review. *Am J Hematol* **86**, 18-24, doi:10.1002/ajh.21904 (2011).
40 Nuijten, M. *et al.* Effectiveness of bevacizumab- and pemetrexed-cisplatin treatment for patients with advanced non-squamous non-small cell lung cancer. *Lung Cancer* **69**, S4–S10 (2010).
41 Riemsma, R. *et al.* Systematic review of aromatase inhibitors in the first-line treatment for hormone sensitive advanced or metastatic breast cancer. *Breast Cancer Res Treat* **123**, 9-24, doi:10.1007/s10549-010-0974-0 (2010).
42 Riemsma, R., Simons, J. P., Bashir, Z., Gooch, C. L. & Kleijnen, J. Systematic Review of topotecan (Hycamtin) in relapsed small cell lung cancer. *BMC Cancer* **10**, 436, doi:10.1186/1471-2407-10-436 (2010).
43 Vissers, D., Stam, W., Nolte, T., Lenre, M. & Jansen, J. Efficacy of intranasal fentanyl spray versus other opioids for breakthrough pain in cancer. *Curr Med Res Opin* **26**, 1037-1045, doi:10.1185/03007991003694340 (2010).
44 Germani, G. *et al.* Clinical outcomes of radiofrequency ablation, percutaneous alcohol and acetic acid injection for hepatocelullar carcinoma: A meta-analysis. *Journal of Hepatology* **52**, 380-388 (2010).
45 Lim, E. *et al.* Preoperative versus Postoperative Chemotherapy in Patients with Resectable Non-small Cell Lung Cancer. *Journal of Thoracic Oncology Official Publication of the International Association for the Study of Lung Cancer* **4**, 1380-1388 (2009).
46 Golfinopoulos, V. *et al.* Comparative survival with diverse chemotherapy regimens for cancer of unknown primary site: multiple-treatments meta-analysis. *Cancer Treat Rev* **35**, 570-573, doi:10.1016/j.ctrv.2009.05.005 (2009).
47 Pignon, J. P., le Maitre, A., Maillard, E., Bourhis, J. & Group, M.-N. C. Meta-analysis of chemotherapy in head and neck cancer (MACH-NC): an update on 93 randomised trials and 17,346 patients. *Radiother Oncol* **92**, 4-14, doi:10.1016/j.radonc.2009.04.014 (2009).
48 Mills, E. J., Rachlis, B., O'Regan, C., Thabane, L. & Perri, D. Metastatic renal cell cancer treatments: an indirect comparison meta-analysis. *BMC Cancer* **9**, 34, doi:10.1186/1471-2407-9-34 (2009).
49 Mauri, D., Polyzos, N. P., Salanti, G., Pavlidis, N. & Ioannidis, J. P. Multiple-treatments meta-analysis of chemotherapy and targeted therapies in advanced breast cancer. *J Natl Cancer Inst* **100**, 1780-1791, doi:10.1093/jnci/djn414 (2008).
50 Sultana, A. *et al.* Gemcitabine based combination chemotherapy in advanced pancreatic cancer-indirect comparison. *BMC Cancer* **8**, 192, doi:10.1186/1471-2407-8-192 (2008).
51 Coleman, C. I., Baker, W. L., Kluger, J. & White, C. M. Antihypertensive medication and their impact on cancer incidence: a mixed treatment comparison meta-analysis of randomized controlled trials. *Journal of Hypertension* **26**, 622-629 (2008).

52 Swart, A. M., Burdett, S., Ledermann, J., Mook, P. & Parmar, M. K. Why i.p. therapy cannot yet be considered as a standard of care for the first-line treatment of ovarian cancer: a systematic review. *Ann Oncol* **19**, 688-695, doi:10.1093/annonc/mdm518 (2008).

53 Golfinopoulos, V., Salanti, G., Pavlidis, N. & Ioannidis, J. P. Survival and disease-progression benefits with treatment regimens for advanced colorectal cancer: a meta-analysis. *Lancet Oncology* **8**, 898-911 (2007).

54 Kyrgiou, M., Salanti, G., Pavlidis, N., Paraskevaidis, E. & Ioannidis, J. P. Survival benefits with diverse chemotherapy regimens for ovarian cancer: meta-analysis of multiple treatments. *J Natl Cancer Inst* **98**, 1655-1663, doi:10.1093/jnci/djj443 (2006).

55 Thompson Coon, J. S. *et al.* Sunitinib and bevacizumab for first-line treatment of metastatic renal cell carcinoma: a systematic review and indirect comparison of clinical effectiveness. *Br J Cancer* **101**, 238-243, doi:10.1038/sj.bjc.6605167 (2009).

56 Zhang, Y. W. *et al.* Chemotherapy for patients with gastric cancer after complete resection: a network meta-analysis. *World J Gastroenterol* **20**, 584-592, doi:10.3748/wjg.v20.i2.584 (2014).

57 Yang, Q. *et al.* Indirect comparison showed survival benefit from adjuvant chemoradiotherapy in completely resected gastric cancer with d2 lymphadenectomy. *Gastroenterol Res Pract* **2013**, 634929, doi:10.1155/2013/634929 (2013).

58 Alba, E. *et al.* Cost-utility analysis of nanoparticle albumin-bound paclitaxel versus paclitaxel in monotherapy in pretreated metastatic breast cancer in Spain. *Expert Review of Pharmacoeconomics & Outcomes Research* **13**, 381-391 (2014).

59 Woods, B., Paracha, N., Scott, D. A. & Thatcher, N. Raltitrexed plus cisplatin is cost-effective compared with pemetrexed plus cisplatin in patients with malignant pleural mesothelioma. *Lung Cancer* **75**, 261-267, doi:10.1016/j.lungcan.2011.07.011 (2012).

60 Ahn, M. J. *et al.* Cost-effectiveness of bevacizumab-based therapy versus cisplatin plus pemetrexed for the first-line treatment of advanced non-squamous NSCLC in Korea and Taiwan. *Asia Pac J Clin Oncol* **7 Suppl 2**, 22-33, doi:10.1111/j.1743-7563.2011.01399.x (2011).

61 Chan, K. *et al.* A Bayesian meta-analysis of multiple treatment comparisons of systemic regimens for advanced pancreatic cancer. *PLoS One* **9**, e108749, doi:10.1371/journal.pone.0108749 (2014).

62 Chen, Y. P. *et al.* A Bayesian network meta-analysis comparing concurrent chemoradiotherapy followed by adjuvant chemotherapy, concurrent chemoradiotherapy alone and radiotherapy alone in patients with locoregionally advanced nasopharyngeal carcinoma. *Ann Oncol* **26**, 205-211, doi:10.1093/annonc/mdu507 (2015).

63 Edwards, S. J., Barton, S., Thurgar, E. & Trevor, N. Topotecan, pegylated liposomal doxorubicin hydrochloride, paclitaxel, trabectedin and gemcitabine for advanced recurrent or refractory ovarian cancer: a systematic review and economic evaluation. *Health Technol Assess* **19**, 1-480, doi:10.3310/hta19070 (2015).

64 Elmunzer, B. J. *et al.* Comparing the effectiveness of competing tests for reducing colorectal cancer mortality: a network meta-analysis. *Gastrointest Endosc* **81**, 700-709 e703, doi:10.1016/j.gie.2014.10.033 (2015).

65 Generali, D. *et al.* A network meta-analysis of everolimus plus exemestane versus chemotherapy in the first- and second-line treatment of estrogen receptor-positive metastatic breast cancer. *Breast Cancer Res Treat* **152**, 95-117, doi:10.1007/s10549-015-3453-9 (2015).

66 Gresham, G. K., Wells, G. A., Gill, S., Cameron, C. & Jonker, D. J. Chemotherapy regimens for advanced pancreatic cancer: a systematic review and network meta-analysis. *Bmc Cancer* **14**, 177-184 (2014).

67 Tejpal Gupta, M. D., Kannan, S., Sarbani Ghosh–Laskar, M. D. & Agarwal, J. P. Concomitant chemoradiotherapy versus altered fractionation radiotherapy in the radiotherapeutic management of locoregionally advanced head and neck squamous cell carcinoma: An adjusted indirect comparison meta-analysis. *Head & Neck* **37**, 670–676 (2014).

68 Haspinger, E. R. *et al.* Is there evidence for different effects among EGFR-TKIs? Systematic review and meta-analysis of EGFR tyrosine kinase inhibitors (TKIs) versus chemotherapy as first-line treatment for patients harboring EGFR mutations. *Crit Rev Oncol Hematol* **94**, 213-227, doi:10.1016/j.critrevonc.2014.11.005 (2015).

69 Huttner, F. J. *et al.* Meta-analysis of reconstruction techniques after low anterior resection for

rectal cancer. *Br J Surg* **102**, 735-745, doi:10.1002/bjs.9782 (2015).
70 Kelly, M. E. *et al.* Synchronous colorectal liver metastasis: a network meta-analysis review comparing classical, combined, and liver-first surgical strategies. *J Surg Oncol* **111**, 341-351, doi:10.1002/jso.23819 (2015).
71 Ladyzynski, P., Molik, M. & Foltynski, P. A network meta-analysis of progression free survival and overall survival in first-line treatment of chronic lymphocytic leukemia. *Cancer Treat Rev* **41**, 77-93, doi:10.1016/j.ctrv.2014.11.004 (2015).
72 Leung, H. W., Chan, A. L. & Lin, S. J. Indirect comparisons of efficacy and safety between seven newer targeted agents for metastatic renal cell carcinoma: A network meta-analysis of randomised clinical trials. *Mol Clin Oncol* **2**, 858-864, doi:10.3892/mco.2014.323 (2014).
73 Liang, W. *et al.* Network meta-analysis of erlotinib, gefitinib, afatinib and icotinib in patients with advanced non-small-cell lung cancer harboring EGFR mutations. *PLoS One* **9**, e85245, doi:10.1371/journal.pone.0085245 (2014).
74 Messori, A., Fadda, V., Maratea, D. & Trippoli, S. First-line treatments for chronic lymphocytic leukaemia: interpreting efficacy data by network meta-analysis. *Ann Hematol* **94**, 1003-1009, doi:10.1007/s00277-015-2310-6 (2015).
75 Nagayama, A. *et al.* Comparative effectiveness of neoadjuvant therapy for HER2-positive breast cancer: a network meta-analysis. *J Natl Cancer Inst* **106**, doi:10.1093/jnci/dju203 (2014).
76 Panagiotopoulou, N. *et al.* Uterine-sparing minimally invasive interventions in women with uterine fibroids: a systematic review and indirect treatment comparison meta-analysis. *Acta Obstet Gynecol Scand* **93**, 858-867, doi:10.1111/aogs.12441 (2014).
77 Pilkington, G. *et al.* A systematic review of the clinical effectiveness of first-line chemotherapy for adult patients with locally advanced or metastatic non-small cell lung cancer. *Thorax* **70**, 359-367, doi:10.1136/thoraxjnl-2014-205914 (2015).
78 Police, R. L. *et al.* Randomized controlled trials in relapsed/refractory chronic lymphocytic leukemia: a systematic review and meta-analysis. *Clin Lymphoma Myeloma Leuk* **15**, 199-207, doi:10.1016/j.clml.2014.09.008 (2015).
79 Popat, S. *et al.* Afatinib in the treatment of EGFR mutation-positive NSCLC--a network meta-analysis. *Lung Cancer* **85**, 230-238, doi:10.1016/j.lungcan.2014.05.007 (2014).
80 Popat, S. *et al.* Nintedanib plus docetaxel as second-line therapy in patients with non-small-cell lung cancer: a network meta-analysis. *Future Oncol* **11**, 409-420, doi:10.2217/fon.14.290 (2015).
81 Sekine, L. *et al.* Conventional and high-dose daunorubicin and idarubicin in acute myeloid leukaemia remission induction treatment: a mixed treatment comparison meta-analysis of 7258 patients. *Hematol Oncol* **33**, 212-219, doi:10.1002/hon.2173 (2015).
82 Sheng, Z. & Zhang, Y. EGFR-TKIs combined with chemotherapy versus EGFR-TKIs single agent as first-line treatment for molecularly selected patients with non-small cell lung cancer. *Med Oncol* **32**, 420, doi:10.1007/s12032-014-0420-y (2015).
83 Tan, P. S., Haaland, B., Montero, A. J., Kyriakopoulos, C. E. & Lopes, G. Hormonal Therapeutics Enzalutamide and Abiraterone Acetate in the Treatment of Metastatic Castration-Resistant Prostate Cancer (mCRPC) Post-docetaxel-an Indirect Comparison. *Clin Med Insights Oncol* **8**, 29-36, doi:10.4137/CMO.S13671 (2014).
84 Tang, J., Zhang, H., Yan, J. & Shao, R. Indirect comparison of the efficacy and safety of gefitinib and cetuximab-based therapy in patients with advanced non-small-cell lung cancer. *Mol Clin Oncol* **3**, 145-150, doi:10.3892/mco.2014.424 (2015).
85 Wang, J.-C., Tian, J.-H., Ge, L., Gan, Y.-H. & Yang, K.-H. Which is the Best Chinese Herb Injection Based on the FOLFOX Regimen for Gastric Cancer? A Network Meta-analysis of Randomized Controlled Trials. *Asian Pacific Journal of Cancer Prevention* **15**, 4795-4800, doi:10.7314/apjcp.2014.15.12.4795 (2014).
86 Wang, Z. *et al.* Systematic literature review and network meta-analysis comparing bone-targeted agents for the prevention of skeletal-related events in cancer patients with bone metastasis. *Oncologist* **20**, 440-449, doi:10.1634/theoncologist.2014-0328 (2015).
87 Xiong, T. *et al.* Comparative efficacy and safety of treatments for localised prostate cancer: an application of network meta-analysis. *Bmj Open* **4**, e004285-e004285 (2014).
88 Yan, M., Kumachev, A., Siu, L. L. & Chan, K. K. Chemoradiotherapy regimens for locoregionally advanced nasopharyngeal carcinoma: A Bayesian network meta-analysis. *Eur J Cancer* **51**,

1570-1579, doi:10.1016/j.ejca.2015.04.027 (2015).
89 Yu, Q., Zhu, Z., Liu, Y., Zhang, J. & Li, K. Efficacy and Safety of HER2-Targeted Agents for Breast Cancer with HER2-Overexpression: A Network Meta-Analysis. *PLoS One* **10**, e0127404, doi:10.1371/journal.pone.0127404 (2015).
90 Zhang, Y. *et al.* Patients with Exon 19 Deletion Were Associated with Longer Progression-Free Survival Compared to Those with L858R Mutation after First-Line EGFR-TKIs for Advanced Non-Small Cell Lung Cancer: A Meta-Analysis. *Plos One* **32**, 294-300 (2013).
91 Zhu, G. Q. *et al.* Systematic review with network meta-analysis: adjuvant therapy for resected biliary tract cancer. *Aliment Pharmacol Ther* **40**, 759-770, doi:10.1111/apt.12900 (2014).
92 Zhu, Z. *et al.* Efficacy and toxicity of external-beam radiation therapy for localised prostate cancer: a network meta-analysis. *Br J Cancer* **110**, 2396-2404, doi:10.1038/bjc.2014.197 (2014).
93 Jiang, Z. M. *et al.* Indirect comparison of different adjuvant chemotherapies for stage Ⅱ-Ⅲ gastric cancer after D2 gastrectomy in Asian patients. *Chin J Gastrointest* **16** (2013). (Chinese)
94 Wei, Y. *et al.* Fluorouracil plus cisplatin combined with trastuzumab or anthracyclines as a first-line therapy for advanced gastric cancer: an indirect comparison based on published literatures. *Chin Arch Gen Surg* **6**, 60-64 (2012).Chinese
95 Kang, Q. & Yu, Z. Indirect Comparisons for the Efficacy of Erlotinib and Pemetrexed in Patients with Nonsmall Cell Lung Cancer. *Drugs and Clinic*, 24-24 (2014).Chinese
96 Tian, J. H. *Network Meta-analyses of Chinese Herb Injection Combined with Chemotherapy for Non-small Cell Lung Cancer*, Lanzhou University, (2012). Chinese
97 Zhao, Y., Liu, D. L., Li, J. L., Ge, L. & Tian, J. H. Chinese Herb Injections Combined with Paclitaxel Plus Cisplatin /Carboplatin for Non-small Cell Lung Cancer: A Network Meta-analysis. *Chin J Drug Evaluation*, 295-299 (2014).Chinese
98 Tian, J. H, Zhao, Y., Li, J. L., Ge, L. & Yang, K. H. Network Meta-analysis of 12 Chinese Herb Injections Combined with Gemcitabine and Cisplatin for Non-small Cell Lung Cancer. *Chin J Drug Evaluation*, 350-355 (2014). Chinese
99 Tian, J. H, Zhao, Y., Li, J. L., Ge, L. & Yang, K. H. Network Meta-analysis of 10 Chinese Herb Injections Combined with Vinorelbine and Cisplatin for Non Small Cell Lung Cancer. *Chin J Drug Evaluation*, 45-49 (2015). Chinese
100 Zhang, Z. *The efficacy of Combination therapy of TACE,PEI,or RFA for hepatocellular carcinoma: meta analysis of direct and indirect evidence*, Central South University, (2014).
101 Wang, J. C. *Network Meta-analyses of Chinese Herb Injection Combined with Chemotheropy for Gastric Cancer*, Lanzhou University, (2014). Chinese
102 Wu, Z. C., Lin, S. L., Luo, Q. H., Cai C.H. & Hong, R. J. Pharmacoeconomics Analysis of Chinese Herb Injection Combined with NP Chemotherapy in Treatment of Non-small Cell Lung Cancer. *Chinese Journal of Experimental Traditional Medical Formulae*, 199-202 (2015). Chinese
